# Characterization of full-length transcriptome in *Saccharum officinarum* and molecular insights into tiller development

**DOI:** 10.1186/s12870-021-02989-5

**Published:** 2021-05-22

**Authors:** Haifeng Yan, Huiwen Zhou, Hanmin Luo, Yegeng Fan, Zhongfeng Zhou, Rongfa Chen, Ting Luo, Xujuan Li, Xinlong Liu, Yangrui Li, Lihang Qiu, Jianming Wu

**Affiliations:** 1 Sugarcane Research Institute of Guangxi Academy of Agricultural Sciences, Guangxi Key Laboratory of Sugarcane Genetic Improvement, and Key Laboratory of Sugarcane Biotechnology and Genetic Improvement (Guangxi), Ministry of Agriculture, East Daxue Road 172, Nanning, 530004 Guangxi China; 2 Sugarcane Research Institute of Yunnan Academy of Agricultural Sciences, East Lingquan Road 172, Kaiyun, 661600 Yunnan China

**Keywords:** Crop productivity, Genomic data, C_4_ plant, Carbon fixation, Linoleic acid, Gene expression

## Abstract

**Background:**

Although extensive breeding efforts are ongoing in sugarcane (*Saccharum officinarum* L.), the average yield is far below the theoretical potential. Tillering is an important component of sugarcane yield, however, the molecular mechanism underlying tiller development is still elusive. The limited genomic data in sugarcane, particularly due to its complex and large genome, has hindered in-depth molecular studies.

**Results:**

Herein, we generated full-length (FL) transcriptome from developing leaf and tiller bud samples based on PacBio Iso-Seq. In addition, we performed RNA-seq from tiller bud samples at three developmental stages (T0, T1 and T2) to uncover key genes and biological pathways involved in sugarcane tiller development. In total, 30,360 and 20,088 high-quality non-redundant isoforms were identified in leaf and tiller bud samples, respectively, representing 41,109 unique isoforms in sugarcane. Likewise, we identified 1063 and 1037 alternative splicing events identified in leaf and tiller bud samples, respectively. We predicted the presence of coding sequence for 40,343 isoforms, 98% of which was successfully annotated. Comparison with previous FL transcriptomes in sugarcane revealed 2963 unreported isoforms. In addition, we characterized 14,946 SSRs from 11,700 transcripts and 310 lncRNAs. By integrating RNA-seq with the FL transcriptome, 468 and 57 differentially expressed genes (DEG) were identified in T1vsT0 and T2vsT0, respectively. Strong up-regulation of several pyruvate phosphate dikinase and phosphoenolpyruvate carboxylase genes suggests enhanced carbon fixation and protein synthesis to facilitate tiller growth. Similarly, up-regulation of linoleate 9S-lipoxygenase and lipoxygenase genes in the linoleic acid metabolism pathway suggests high synthesis of key oxylipins involved in tiller growth and development.

**Conclusions:**

Collectively, we have enriched the genomic data available in sugarcane and provided candidate genes for manipulating tiller formation and development, towards productivity enhancement in sugarcane.

**Supplementary Information:**

The online version contains supplementary material available at 10.1186/s12870-021-02989-5.

## Background

Sugarcane (*Saccharum officinarum* L.) is an important economic crop of the grass family. It is cultivated in the tropic and subtropic regions and represents the main source of world’s sucrose [1]. Biofuel, fiber, fertilizer and several other byproducts are also derived from sugarcane production [2]. It is estimated that more than 45 million farmers are involved in the sugarcane production worldwide and nearly 2 billion tonne of sugarcane were produced in 2019 [1]. Despite an extensive breeding, worldwide sugarcane average yield (84 t/ha) is far below the theoretical potential (384 t/ha), therefore, considerable efforts are still needed to increase the crop productivity [3].

Sugarcane can be harvested for many years and the plant is mainly composed of stalks, which are derived from tillers. Similar to other grasses, a single sugarcane plant can produce multiple stalks [4]. Tillering is the sprouting of lateral buds, which can subsequently develop into mature stalks, therefore it is an important component of sugarcane yield. Elucidating the molecular mechanism of tiller development is critical for sugarcane productivity.

Tillering has early catalyzed attention of researchers. In rice, the cloning and functional identification of the gene *MOC1* has marked a breakthrough in the tillering regulation mechanism [5]. Later on, several genes such as *MOC3*/*TAB1*/*SRT1*, *LAX1*, *LAX2, FON1*, *SLR1* and *TAD1* have been reported to coordinately interact with *MOC1* for tiller formation and development in rice [5–13]. It has been reported that *TaD27-B* gene controls tiller number in hexaploid wheat by regulating strigolactone content [14]. In maize, a complex gene regulatory network involving *tb1*, *gt1*, *tru1*, *sugary1* and *tin1* controls tiller development [15–19]. Collectively, it has become evident that at the genome level, tillering is a multigenic trait underlined by fine coordination of the expression of many genes involved in various biological pathways such as cell cycle, growth and development and phytohormone signaling [20, 21]. In sugarcane, no specific gene controlling tiller development has been documented so far. More importantly, the molecular mechanism of tiller development in sugarcane is still elusive.

Genomics-assisted breeding has become a revolutionary strategy for crop improvement and has high potentials for sugarcane improvement [22]. However, it requires high-quality genomic resources such as complete genome sequences, re-sequencing and gene expression data. Sugarcane modern cultivars (*Saccharum spp*, 2n = 100–120) are heteropolyploid with a large (~ 10 Gb) and highly complex genome [23]. Several initiatives have been launched to generate genomics resources in sugarcane, particularly, for developing a high-quality reference genome. This resulted in the release of several genome sequences, most of them being of low-quality, fragmented and incomplete [24–29]. In addition, several transcriptome data based on next generation RNA-seq technologies were generated in sugarcane, providing important gene reservoirs for functional studies [3]. Recently, third generation sequencing technologies such as single molecule real-time (SMRT) sequencing developed by PacBio (Pacific Biosciences of California, Menlo Park, CA, USA) are used for transcriptome studies [30]. SMRT generates high quality full-length (FL) transcripts and facilitates the identification of isoforms and repeat regions in the genome. For species without a good reference genome sequence, SMRT could further enrich the available genomic resources through the reconstruction of the coding genome [31, 32].

The present study aimed at reconstructing the FL transcriptome of sugarcane and investigating the molecular basis of tiller development. To achieve this objective, we prepared two separate PacBio Iso-Seq libraries from developing leaf and axillary tiller bud tissues of sugarcane seedlings to reconstruct and characterize the FL transcriptome. In addition, we performed RNA-seq from axillary tiller bud tissues at three developmental stages to uncover differentially expressed genes and biological pathways underlying tiller development in sugarcane.

## Results

### Construction and annotation of *S. officinarum* full-length transcriptome

**Fig. 1:**
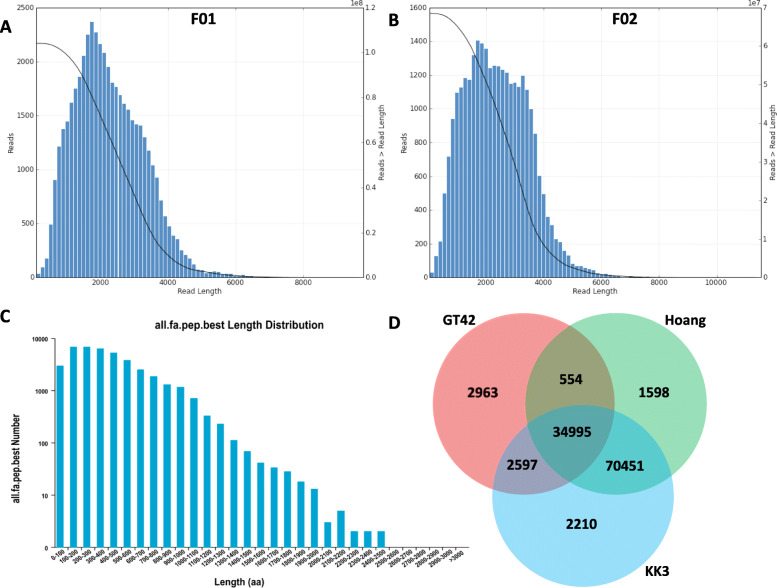
Overview of full-length transcriptome sequencing using PacBio Sequel platform in *S. officinarum*. **a** The length distribution of reads in F01 representing the leaf library. The horizontal axis represents the length, the vertical axis represents the number of reads within the length range; **b** The length distribution of reads in F02 representing the tiller bud library; **c** CDS length distribution in the two libraries; **d** Venn diagram depicting the shared and specific number of FLNC in three Iso-Seq transcriptome datasets. Hoang, KK3 and GT42 refer to Hoang et al. [31], Piriyapongsa et al. [32] and the current study, respectively

We constructed two SMRT libraries for leaf samples (F01) and tiller bud samples (F02). Libraries were sequenced each with 3 cells, yielding 19,7 and 23,63 Gb clean data for F01 and F02, respectively. A total of 570,055 and 357,140 CCS were identified in F01 and F02, respectively, and classified as FL based on the presence of 5′ primers, 3′ primers and poly(A) tail (Table 1). The distribution of transcript lengths ranged from 150 to 8000 and 200 to 10,000 bp in F01 and F02, respectively (Fig. 1a, b). After, polishing using RNA-Seq reads, clustering and demultiplexing of FL transcripts, 30,360 and 20,088 high-quality non-redundant FLNC were identified in F01 and F02, respectively (Table 2). We identified 1063 and 1037 AS events in F01 and F02, respectively (Table S1; S2). By merging FLNC transcript lists from the two libraries, we identified 41,109 unique FLNC transcripts in *S. officinarum* spanning 91,227,518 bp. A total of 40,343 CDSs were predicted in the FL transcriptome with length ranging from 100 to 2500 bp (Fig. 1c). Functional annotation of the FLNC transcripts was conducted using eight different public databases and 39,581 transcripts were successfully annotated in at least one database (Table 3; Table S3). These annotated transcripts were grouped into 3640 gene families (Table S4), including 1166 PK, 1134 TF and 324 TR genes (Table S5).

**Table 1:** PacBio sequencing data statistics

Samples	Library	cDNA size (kb)	CCS Number	Read Bases of CCS (bp)	Mean Read Length of CCS (bp)	Mean Number of Passes
Leaf	F01	1–6	570,055	1,407,018,638	2468	19
Tiller bud	F02	1–6	357,140	934,768,566	2617	25

**Table 2:** Processing of PacBio data and identification of FLNC

Samples	Library	Number of undesired primer reads	Number of undesired poly-A reads	Number of filtered short reads	Number of full-length nonchimeric Reads (FLNC)	Non redundant FLNC
Leaf	F01	61,250	413,783	309	475,033	30,360
Tiller bud	F02	37,436	266,227	190	303,663	20,088

**Table 3:** Annotation statistics of the FLNC

Annotation database	Annotated number	300 < =length (bp) < 1000	Length (bp) > =1000
COG	16,596	1370	15,221
GO	33,953	3495	30,448
KEGG	17,198	1897	15,287
KOG	24,191	2071	22,113
Pfam	33,970	3173	30,793
Swissprot	29,225	2695	26,519
eggNOG	38,644	4069	34,557
nr	39,491	4263	35,200
All annotated	39,581	4311	35,242

We compared the unique FLNC obtained in this study with previous FL transcriptome sequencing in sugarcane [31, 32]. We observed that 85% of the FLNC identified in this study was conserved among other sugarcane cultivars while 2963 FLNC did not match previous Iso-Seq study of sugarcane (Fig. 1d).

### Characterizations of SSRs and lncRNAs

**Fig. 2:**
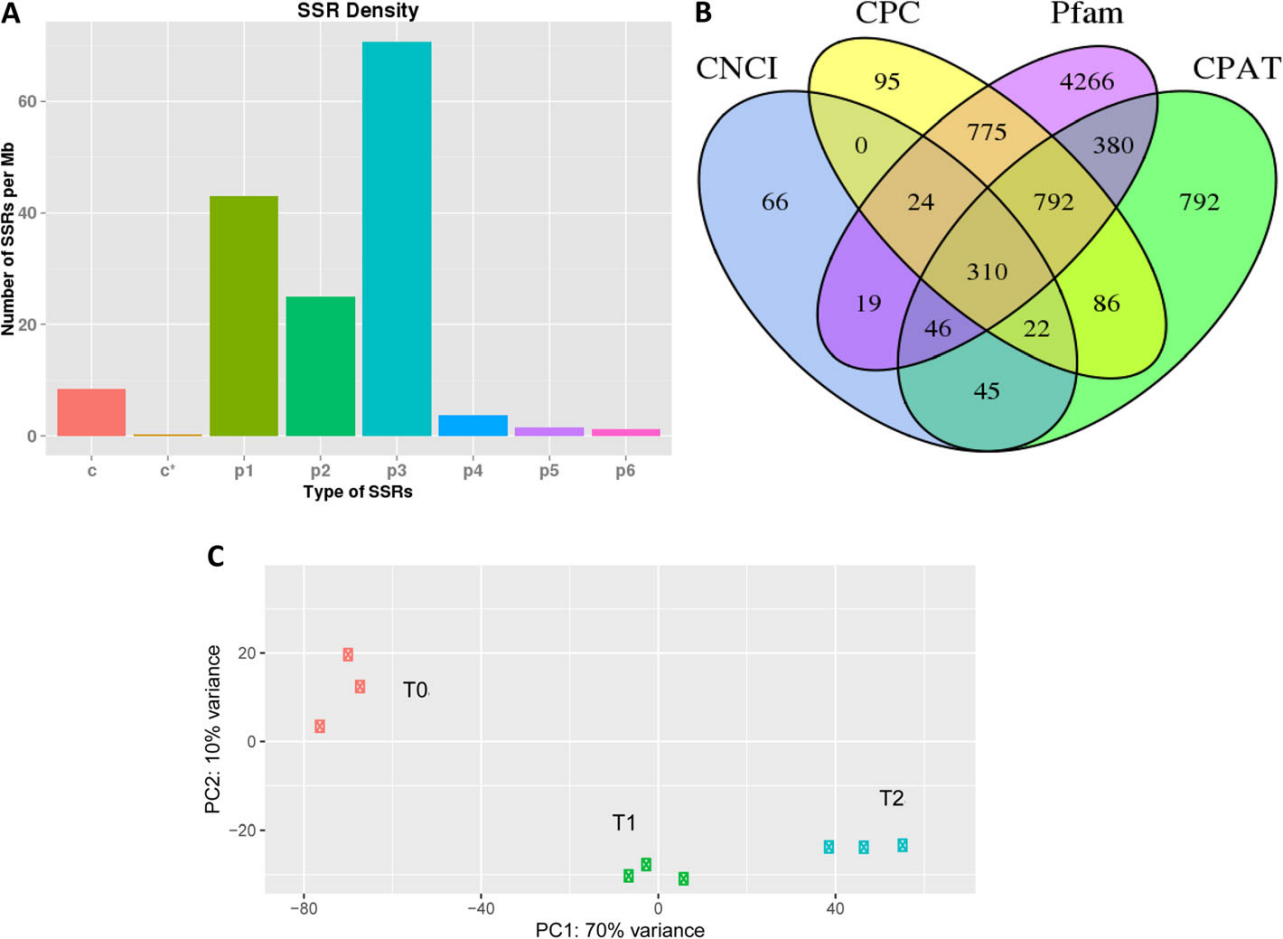
Identification of simple sequence repeat (SSR) and lncRNA and in *S. officinarum* full-length transcriptome and characterization of Illumina short read transcriptome. **a** Statistics and characteristics of the SSRs detected. X axis represents the SSR types and Y axis is the number of SSRs per Mb; **b** Venn diagram showing the number of shared and specific detected lncRNA using CNCI, CPAT, Pfam and CPC programs; **c** Principal component analysis based on FPKM data from *S. officinarum* Illumina short read transcriptome of tiller bud tissues collected at three growth stages (T0, T1 and T2). c*,c = compound SSRs; p1-p6 = mono-, di-nucleotide, tri-nucleotide, tetra-nucleotide, penta-nucleotide, and hexa-nucleotide

We also examined the presence of SSRs and lncRNAs in the FL transcriptome of *S. officinarum*. We identified 14,946 SSRs in 11,700 transcripts, dominated by tri- and mono-nucleotide SSR types (Fig. 2a; Table S6). A total of 2535 genes contained more than 1 SSR and 879 compound SSRs were detected. With regard to lncRNAs, we detected 310 lncRNAs conserved among the four tools used for lncRNA prediction (CPC, CNCI, Pfam, and CPAT) (Fig. 2b; Table S7).

### Short-reads transcriptome

RNAs extracted from tiller bud samples collected at T0, T1 and T2 in three biological replicates, were used for RNA-seq analysis based on the Illumina HiSeq Ten X platform. In total, nine RNAs were sequenced, yielding more than 500 million raw short-reads (Table S8). After filtering out low-quality reads, we obtained ~ 79 Gb clean data representing ~ 99% of the raw data. On average, the clean reads had a Q30 score of ~ 93% and a GC content of ~ 54%, showing a high quality of the sequencing data. The clean reads were mapped to *S. officinarum* FL transcriptome and gene expression was estimated using the FPKM method. We performed a principal component analysis (PCA) based on FPKM data to assess the pattern of clustering of biological replicates and samples. As shown in Fig. 2c, all biological replicates were clustered together, indicating a high correlation between them. In addition, we observed a clear separation of the tiller bud samples collected from the three growth stages. These results suggest significant effects of growth stages on *S. officinarum* tiller bud transcriptome.

### Differentially expressed genes (DEG) in tiller bud tissues over growth stages

**Fig. 3:**
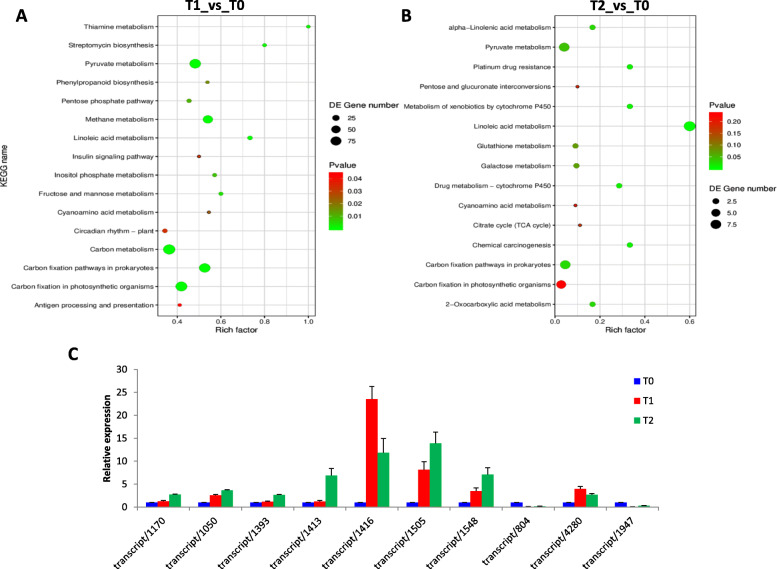
KEGG enrichment analysis of the DEGs identified between **a** T1 and T0, **b** T2 and T0; **c** qRT-PCR validation of ten selected genes. The x-axis represents the ten genes while the y-axis represents the relative expression of each gene. The bars show standard deviation

Gene expression profiles of tiller bud samples at T0 were compared with T1 and T2 in order to identify DEGs. We identified 468 DEGs (369 up- and 99 down-regulated) in T1vsT0 while only 57 DEGs (40 up- and 17 down-regulated) were obtained in T2vsT0. KEGG enrichment analysis of the DEGs in T1vsT0 showed carbon fixation pathways and pyruvate metabolism as the most enriched biological pathways (Fig. 3a). Concerning the comparison T2vsT0, we observed that carbon fixation pathways, linoleic acid metabolism and pyruvate metabolism were the most enriched KEGG pathways (Fig. 3b).

We randomly selected ten DEGs and quantified their transcript levels using qRT-PCR method (Table S9). The gene *Actin* was used as endogenous control for gene expression normalization. The results showed that transcript levels of all selected genes were significantly altered at T1 and T2 as compared to T0 similarly as observed in the RNA-seq data (Fig. 3c). These observations indicate the reliability of the DEG analysis conducted in this study.

### Carbon fixation pathways

**Fig. 4:**
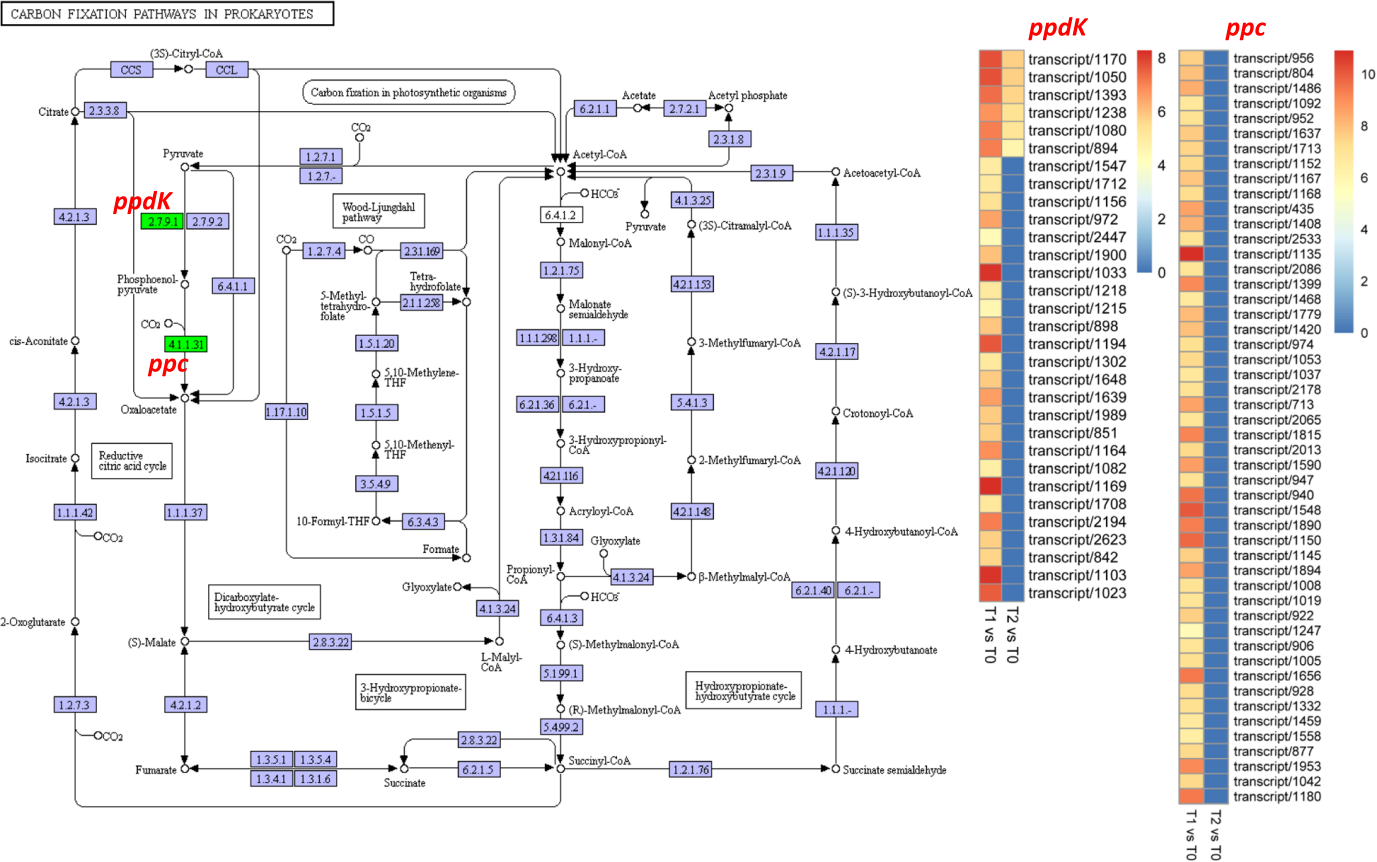
Carbon fixation pathway elaborating the DEG between transcriptomes of *S. officinarum* tiller bud samples collected at three growth stages. Green boxes show the DEGs while blue boxes represent genes expressed in tiller bud samples but not differentially expressed. Heatmaps illustrate the expression pattern of genes between T1 vs T0 and T2 vs T0

Carbon fixation in plants is the process by which inorganic carbon is converted to organic compounds. In this study, 81 DEGs mapped to the carbon fixation pathways were screened out. Interestingly, all these DEGs were annotated as pyruvate phosphate dikinase (*ppdk*) and phosphoenolpyruvate carboxylase (*ppc*). The *ppdks* are involved in the conversion of pyruvate into phosphenol pyruvate, which is subsequently converted to oxaloacetate by *ppcs* (Fig. 4). Oxaloacetate is then reduced to malate, which represents the input of the Calvin cycle. All the 31 *ppdks* were strongly upregulated at T1 and T2 as compared to T0. Similarly, the 50 *ppcs* identified were all up-regulated at T1 as compared to T0. Altogether, these results suggest that *S. officinarum* tiller development is underlined by a strong synthesis of oxaloacetate.

### Linoleic acid metabolism

**Fig. 5:**
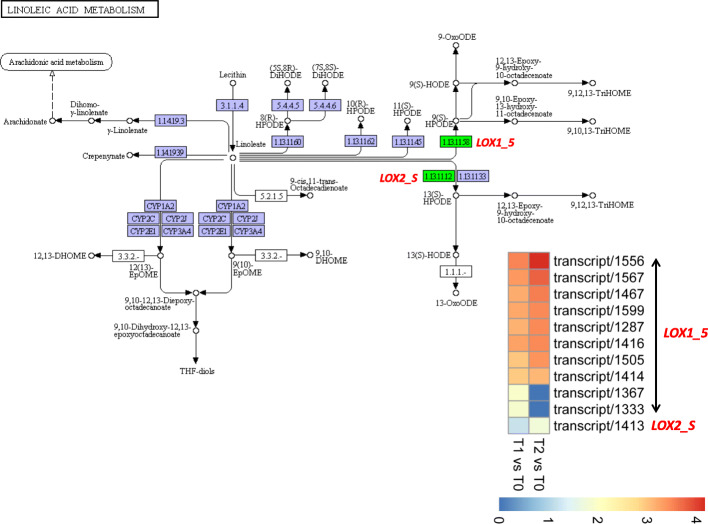
Linoleic acid metabolism pathway elaborating the DEG between transcriptomes of *S. officinarum* tiller bud samples collected at three growth stages. Green boxes show the DEGs while blue boxes represent genes expressed in tiller bud samples but not differentially expressed. Heatmaps illustrate the expression pattern of genes between T1 vs T0 and T2 vs T0

A total of 11 DEGs were mapped to the linoleic acid metabolism pathway, including 10 linoleate 9S-lipoxygenase (*LOX1_5*) and one lipoxygenase (*LOX2_S*) (Fig. 5). *LOX1_5* and *LOX2_S* convert linoleate to (9S)-HPODE and (13S)-HPODE, respectively, which are converted into a large class of oxygenated polyenoic fatty acids called oxylipins [33]. Up-regulation of these genes in T2 and T1 as compared to T0 indicates a mechanism towards high synthesis of oxylipins, which may be crucial for *S. officinarum* tiller growth.

### Transcription factors

Plant transcription factors (TFs) regulate numerous physiological programs important for plant growth and development. In this study, we detected nine DEGs encoding TFs belonging to five different families (Table 4). Most of the TFs were differentially expressed between T1 and T0 and data showed that they were up-regulated. Only the gene *transcript/1947* was down-regulated at T2vsT0. We predict that these TFs play vital roles in regulating structural genes involved in *S. officinarum* tiller development.

**Table 4:** Transcription factors differentially expressed over *S. officinarum* tiller growth stages

Gene ID	T1vsT0 (Log 2 fold change)	T2vsT0 (Log 2 fold change)	TF family
*transcript/1548*	10.14	0.00	B3-ARF
*transcript/3906*	3.85	0.00	B3-ARF
*transcript/4280*	1.97	0.00	B3-ARF
*transcript/313*	1.07	0.00	C3H
*transcript/2433*	1.53	0.00	MYB
*transcript/1947*	0.00	−1.54	MYB
*transcript/3790*	2.40	0.00	RWP-RK
*transcript/2444*	3.27	0.00	RWP-RK
*transcript/2533*	7.00	0.00	WRKY

## Discussion

The largest genomic data in sugarcane has been recently released by Souza et al. [28]. It spanned 4.26 Gb long, representing ~ 30% of the estimated genome size and 373,869 gene models were predicted. Hence, it is obvious that a high proportion of gene models are still unreported in sugarcane. Besides, it is well known that more than 95% of genes experience alternative splicing events, leading to multiple isoforms of each gene [34]. Deciphering the landscape of isoforms in sugarcane transcriptome will provide a useful resource for improving gene model prediction and annotation as adopted in various plant species such as in wheat, maize, panax and sorghum [35–38]. In this study, we employed Pacbio Iso-Seq to generate full-length transcriptome from two sugarcane tissues. In total, 30,360 and 20,088 FLNC transcripts were obtained in leaf and tiller tissues, respectively. The variation of the number of FLNC transcripts in both tissues highlights the tissue specific expression pattern of several genes in sugarcane, which could associate with important agronomic traits and thus provide new marker tools for breeding programs [32]. Compared with previous FL transcriptome sequencing in sugarcane [31, 32], we obtained the lowest number of FLNC transcripts. This can be explained by several factors such as the tissues sampled, the genotype, the growth stage and even the PacBio transcriptome sequencing and data processing procedures. For example, Hoang et al. [31], pooled RNA from internode, leaf and root collected at different developmental stages in 22 sugarcane varieties. They reported 107,598 unique transcripts which is more than the double of the number of FLNC transcripts identified in this study. Nonetheless, we obtained the highest number of specific FLNC transcripts in this study. Notably, there is no previous report of axillary tiller bud tissue transcriptome in sugarcane, hence, our results provide new catalog of useful transcripts for improving genome annotation in sugarcane.

Beyond being advantageous to recover full-length transcripts, PacBio Iso-Seq allows identification of lncRNAs and repetitive sequences such as SSRs [39]. Growing evidences demonstrate that lncRNAs are key regulators of gene expression and genome stability in plants and are involved in functions such as vernalization, fertility, photomorphogenesis, phosphate homeostasis, protein re-localization, modulation of chromatin loop dynamics [40, 41]. We reported here 310 lncRNAs with high confidence, which will be instrumental for further illuminating the complex biology of sugarcane. SSRs are the most widely used molecular markers in plant thanks to their relatively abundance, co-dominance, high polymorphism, easy and low-cost procedure [42]. Importantly, it has been demonstrated that transcriptome based SSRs are linked to functional genes, hence they can be used to study their association with phenotypic variation and the flanking sequences are more likely to be conserved among close or distant species, making their use as markers for comparative mapping easier [43]. Currently, a limited number of sugarcane-specific SSR markers are available [44], hence, the 14,946 SSRs detected in our study, pending validation and screening of the most polymorphic ones, will be very useful for genotyping and genetic diversity studies.

By integrating RNA-seq data from axillary tiller bud at three developmental stages with our FL transcriptome, we investigated the DEGs and enriched pathways associated with tiller development in sugarcane seedlings. We obtained higher number of DEGs at T1 vs T0 as compared to T2 vs T0, indicating that major transcriptome readjustment occurs in order to initiate tiller bud germination in *S. officinarum* seedlings. Two key biological pathways were found enriched during tiller development in *S. officinarum* seedlings: carbon fixation and linoleic acid metabolism pathways.

Carbon fixation is the process by which CO_2_ is incorporated into organic compounds [45], which are used to store energy and as building blocks for other important plant biomolecules. It is well known that enhancing carbon fixation is a viable approach for improving plant growth and biomass accumulation [46–48]. Two key gene families were strongly up-regulated in the carbon fixation pathways during tiller development in sugarcane seedlings: pyruvate phosphate dikinase (*ppdk*) and phosphoenolpyruvate carboxylase (*ppc*). In species such as sugarcane that use C_4_ photosynthesis, the actions of these two enzymes lead to synthesis of oxaloacetate, which is reduced to malate, as input of the Calvin cycle [49]. Transgenic plants overexpressing *ppdk* or *ppc* displayed improved carbon assimilation and growth [50–54]. Collectively, we deduced that up-regulation of *ppc* and *ppdk* increases the supply of 4-carbon carboxylic acids, providing high carbon skeletons to sustain high amino acid and protein synthesis responsible for increased metabolic processes and tiller growth in sugarcane seedlings.

Plant lipoxygenases (*LOX*) oxidize polyunsaturated fatty acids such as linolenic and linoleic acids into fatty acid hydroperoxides, which are converted into oxylipins [33]. LOX-derived oxylipins are involved in various physiological processes of plants, including growth and development. For example, Kolomiets et al. [55] demonstrated that *POTLX-1* controls tuber growth and development in potato, probably by initiating the synthesis of oxylipins that regulate cell growth during tuber formation. In Arabidopsis, it has been shown that oxylipins produced by the 9-*LOX* pathway regulate lateral root development [56]. Similarly, the *lox3–4* knockout maize mutants displayed reduced root length and plant height compared with the wild type, indicating that *ZmLOX3* is required for normal plant development Gao et al. [57]. The strong upregulation of *LOX* genes in this study indicates a mechanism towards high synthesis of key oxylipins involved in tiller growth and development in sugarcane. Jasmonic acid (JA) is a type of oxylipins and several studies have demonstrated that JA contents affect tillering in grass [58, 59]. Future studies should investigate developing sugarcane tiller bud samples to clarify whether JA or other types of oxylipins promote tiller development.

## Conclusions

In this study, we enriched the genomic data available in sugarcane by generating and characterizing the full-length transcriptome from developing leaf and tiller bud tissues. The novel transcripts identified will be useful for ongoing efforts of genome annotation and gene model prediction in sugarcane. By integrating RNA-seq data from developing axillary tiller bud tissues, we identified important genes involved in the carbon fixation and linoleic acid pathways differentially expressed during tiller development. Further in-depth investigations of these candidate genes will provide prospects for controlling tiller outgrowth and productivity in sugarcane.

## Methods

### Plant material

In this study, *S. officinarum* L. cultivar Guitang 42 (GT42) was employed as plant material. It is an excellent cultivar with high-yield, high-sugar, lodging-resistance and suitable for mechanized production, developed by Sugarcane Research Institute, Guangxi Academy of Agricultural Sciences, China [60]. Plant material was obtained from Sugarcane Research Institute, Guangxi Academy of Agricultural Sciences, China. The formal identification of the material was undertaken by the corresponding author of this study Prof Lihang Qiu. No voucher of the plant material has been deposited in a genebank. Healthy stems were selected, cut into single bud segments, then washed with water and soaked for 30 min. Thirty stems with only one bud were used as propagules and planted in pots (24 × 19.5 × 26.5 cm), with 3 propagules per pot. Each pot was filled with 11 kg of peat nutrient soil, covered with plastic film and placed in a greenhouse located at the experimental field of Sugarcane Research Institute of Guangxi Academy of Agricultural Sciences (latitude: 22.85, longitude: 108.24, altitude 50.17 m) and the microclimate conditions for sugarcane growth in the greenhouse were as follows: average temperature = 36.52 ± 0.76 °C, average relative humidity = 75.66 ± 2.4% and average light intensity = 1884.1 ± 39.25 μmol m^− 2^ s^− 1^. When the buds sprouted out of the soil, the plastic film was removed, and water was sprayed in each pot to ensure a normal development. Subsequently, samples of sugarcane leaf tissues and tiller buds were taken at different growth stages, including T0 (2–3 leaves stage; establishment stage), T1 (4–5 leaves stage; beginning of tillering stage), and T2 (6–7 leaves stage; full tillering stage). For leaf samples, the first true leaf was collected from the three plants of each pot and mixed to form a biological replicate. For the tiller bud samples, at the T0 the axillary tiller buds start to germinate belowground. At T1, the axillary tiller buds reach about 0.5 cm–1 cm length belowground. At T2, the axillary tiller buds emerge from the soil and the length reaches about 2 cm. At each stage, axillary tiller buds were collected from the three plants of each pot and mixed to form a biological replicate. Samples were collected in three biological replicates from plants grown in different pots and quickly frozen with liquid nitrogen and stored at − 80 °C.

### Library construction and single-molecule real-time (SMRT) sequencing

Total RNA was extracted by grinding mixed leaf samples collected at the three growth stages and mixed tiller bud samples from the three growth stages, separately, in TRIzol reagent (Life technologies) following the manufacturer’s protocol and two libraries were constructed. The integrity of the RNAs was determined with the Agilent 2100 Bioanalyzer (Agilent Technologies, Santa Clara, CA, USA) and agarose gel (1%) electrophoresis. The purity and concentration of the RNAs were determined with the Nanodrop micro-spectrophotometer (Thermo fisher Scientific, Waltham, MA, USA). The mRNA was enriched by Oligo (dT) magnetic beads. Then the enriched mRNA was reverse transcribed into cDNA using Clontech SMARTer PCR cDNA Synthesis Kit (Clontech Laboratories, USA). PCR cycle optimization was used to determine the optimal amplification cycle number for the downstream large-scale PCR reactions. Then, the optimized cycle number was used to generate double-stranded cDNA. Moreover, 1–6 kb size selection was performed using the Blue Pippin TM Size-Selection System. Then, large-scale PCR was performed for the next SMRTbell library construction. cDNAs were repaired for DNA damage and ligated to sequencing adapters. The SMRTbell template was annealed to sequencing primer, bound to polymerase, and sequenced on the PacBio Sequel platform using P6-C4 chemistry with 10 h movies by Biomarker Technology Co. (Beijing, China).

### PacBio long-read processing

PacBio data processing followed standard procedures established by Biomarker Technology Co. (Beijing, China). The raw sequencing reads of cDNA libraries were classified and clustered into transcript consensus using the SMRT Link v5.0.1pipeline [61]. First, circular consensus sequence (CCS) reads were extracted out of subreads BAM file. Next, CCS reads were classified into full-length non-chimeric (FL), non-full-length (nFL), chimeras, and short reads based on cDNA primers and poly A tail signal. Short reads (< 50 bp) were discarded from the analysis. Subsequently, the full-length non-chimeric reads were clustered by Iterative Clustering for Error Correction software to generate the cluster consensus isoforms. To improve accuracy of PacBio reads, two strategies were employed. First, the nFL reads were used to polish the above obtained cluster consensus isoforms by Quiver software [62] to obtain the FL polished high quality consensus sequences (accuracy ≥90%). Next, the low quality isoforms were further corrected using Illumina short reads by using the LoRDEC tool (version 0.8) [63]. Then, the final transcriptome isoform sequences were filtered by removing the redundant sequences with software CD-HIT-v4.6.7 [64] using an identity threshold of 0.99.

### Annotation of genes and identification of transcription factor (TF), transcriptional regulators (TR) and protein kinases (PK)

We used TransDecoder software (https://github.com/TransDecoder/TransDecoder/wiki) for the coding sequence (CDS) prediction. Next, DNA or protein sequences of the FLNC transcripts were submitted to various public databases for functional annotation: clusters of orthologous groups/eukaryotic orthologous groups (COG/KOG) database (http://www.ncbi.nlm.nih.gov/COG), Gene Ontology (GO), NCBI non-redundant protein (Nr), Swiss-Prot protein (Swissprot) database (http://www.expasy.ch/sprot), Kyoto Encyclopedia of Genes and Genomes (KEGG) database (http://www.genome.jp/kegg), evolutionary genealogy of genes: Non-supervised Orthologous Groups (eggNOG) database (http://eggnog5.embl.de/) and Pfam protein families (Pfam) database (https://pfam.xfam.org/). To identify plant transcription factors (TFs), transcriptional regulators (TRs) and protein kinases (PKs), transcripts were submitted to Plant Transcription Factor database (PlantTFdb: http://planttfdb.gao-lab.org/, [65]) and iTAK (v.1.5, [66]) with the best match result [67].

### Simple sequence repeats (SSR) prediction

MIcroSAtellite (MISA, http://pgrc.ipk-gatersleben.de/misa/) is a package that identifies seven types of simple sequence repeat (SSR): mono- nucleotide, di-nucleotide, tri-nucleotide, tetra-nucleotide, penta-nucleotide, hexa-nucleotide and compound SSR (hybrid microsatellite). We screened transcripts with length > 500 bp using the following parameters: unit_size- min_repeats: 2–6, 3–5, 4–4, 5–4, 6–4; compounds (max_difference_between_2_SSRs): 100.

### Characterization of long non-coding RNAs (lncRNAs)

Four tools were used to predict the lncRNAs: CNCI (v.2) [68], CPC (v.0.9-r2) [69]), Pfam protein families (Pfam) database (https://pfam.xfam.org/) and CPAT (v.3.0.0) [70]. In this study, we kept the commonly detected lncRNAs between the four tools as the most probable lncRNAs.

### Alternative splicing (AS) detection

AS transcripts were predicted following methods described by Pan *et al.* [71]. All sequences were run all-vs-all BLAST with high identity settings [72] against the assembled FL transcriptome. BLAST alignments that met all criteria were considered products of candidate AS events: (1) both sequence (query and subject) lengths exceeded 1000 bp and the alignment contained 2 high-scoring segment pairs (HSPs); (2) the alternative splicing gap exceeded 100 bp and was located ≥100 bp from the 3′/5′ end; and (3) a 5-bp overlap was allowed for all alternative transcripts.

### RNA-Seq library construction and sequencing

High-quality RNAs were extracted from tiller buds collected at the three growth stages in triplicate using TRIZOL® reagent (TIANGEN, Beijing, China) according to the manufacturer’s protocol. Extracted RNA from different samples was purified by using RNase-free DNase I (TaKaRa, Kyoto, Japan) to remove the genomic DNA contamination. cDNA library construction and sequencing on Illumina HiSeq Ten X platform were conducted following standard procedures established by Biomarker Technology Co. (Beijing, China) and fully described by Li et al. [73].

### Gene expression quantification and differentially expressed genes (DEG) analysis

Raw RNA-Seq data were processed by the fastQC v0.11.2. Paired-end clean reads were aligned to the reference FL transcriptome using Bowtie2 [74] with default parameters. Gene expression was estimated by the number of fragments per kilobase of the transcript sequence per million base pairs sequenced (FPKM) method. The differential expression analysis was performed using the DESeq2 package. Genes with fold change ≥2 and a false discovery rate (FDR) < 0.05 were considered as significantly differentially expressed genes (DEGs) in comparative analysis. DEGs were subjected to enrichment analysis of KEGG [75] and COG [76] pathways. To validate DEG analysis, ten genes were selected and their expression levels were validated based on qRT-PCR approach in three biological and three technical replicates following descriptions of Dossa et al. [77].

## Supplementary Information

**Additional file 1: Table S1.** Alternative splicing events and isoforms identified in *S. officinarum* full-length transcriptome based on leaf sample library. **Table S2.** Alternative splicing events and isoforms identified in *S. officinarum* full-length transcriptome based on tiller bud sample library. **Table S3.** Gene annotation statistics. **Table S4.** Gene family statistics. **Table S5.** List of genes annotated as transcription factors (TF), transcriptional regulators (TRs) and protein kinases (PK). **Table S6.** Characterization of the simple sequence repeats in *S. officinarum* full-length transcriptome. **Table S7.** Characterization of lncRNAs in *S. officinarum* full-length transcriptome. LncRNAs highlighted in red are those commonly identified by the four software. **Table S8.** Statistics of Illumina RNA-seq in *S. officinarum* tiller bud over three growth stages. **Table S9.** Primer sequences of genes used for qRT-PCR validation of differentially expressed genes.

## Data Availability

The RNA-seq data has been submitted to NCBI SRA: PRJNA723212 and accessible at www.ncbi.nlm.nih.gov/sra/PRJNA723212.

## Abbreviations

DEG: Differentially expressed genes
FL: Full length
FLNC: Full length non coding
PCA: Principal component analysis
FPKM: Fragments Per Kilobase of Transcript per Million Fragments Mapped
TF: Transcription factor
lncRNA: Long non coding RNA
SSR: Simple sequence repeat
